# Safety and pharmacology of AMY109, a long-acting anti–interleukin-8 antibody, for endometriosis: a double-blind, randomized phase 1 trial

**DOI:** 10.1016/j.xfre.2025.04.009

**Published:** 2025-05-02

**Authors:** Peng-Hui Wang, Sheng-Mou Hsiao, Shunji Matsuki, Ryuzo Hanada, Chun-An Chen, Ayako Nishimoto-Kakiuchi, Mayuko Sekiya, Kiyohiko Nakai, Junnosuke Matsushima, Mari Sawada

**Affiliations:** a Department of Obstetrics and Gynecology, Taipei Veterans General Hospital and National Yang Ming Chiao Tung University, Taipei, Taiwan; b Department of Obstetrics and Gynecology, Far Eastern Memorial Hospital, New Taipei, Taiwan; c Department of Clinical Research Center, Souseikai Fukuoka Mirai Hospital, Fukuoka, Japan; d Souseikai Sumida Hospital, Tokyo, Japan; e Translational Research Division, Chugai Pharmaceutical Co., Ltd., Tokyo, Japan; f Drug Safety Division, Chugai Pharmaceutical Co., Ltd., Tokyo, Japan; g Clinical Development Division, Chugai Pharmaceutical Co., Ltd., Tokyo, Japan; h Department of Obstetrics and Gynecology, Kurashiki Medical Center, Okayama, Japan

**Keywords:** AMY109, anti–interleukin-8 antibody, endometriosis, pharmacokinetics, safety

## Abstract

**Objective:**

To evaluate the safety and pharmacokinetics/pharmacodynamics of AMY109, an anti–interleukin-8 recycling antibody in a first-in-human phase 1 trial in healthy volunteers (HVs) and patients with endometriosis.

**Design:**

A multicenter, randomized, double-blind, placebo-controlled single-dose and multiple ascending-dose study in Japan and Taiwan.

**Subjects:**

Asian and White HVs in part 1 and patients with endometriosis aged ≥20 to <50 years in part 2.

**Intervention:**

In part 1, sequential cohorts of HVs randomly received a single subcutaneous dose of AMY109 (0.6, 2.0, 3.5, or 5.0 mg/kg) or placebo. In part 2, sequential cohorts of patients with endometriosis randomly received a once-monthly subcutaneous dose of AMY109 (0.8, 2.0, or 5.0 mg/kg) or placebo for 6 months.

**Main Outcome Measures:**

The primary objective was to assess the safety and tolerability of AMY109. Pharmacokinetic profiles and efficacy were evaluated as secondary and exploratory endpoints, respectively.

**Results:**

Overall, 42.1% (32/76) of HVs in part 1 and 61.5% (16/26) of patients in part 2 experienced at least one adverse event during the study. The most common adverse events were oropharyngeal pain, pharyngitis, upper respiratory tract infections, and upper respiratory tract inflammation in part 1 (≥5.0%) and vomiting, nasopharyngitis, diarrhea, nausea, and vaccination site pain in part 2 (≥15.0%). Most events were mild to moderate in severity and were resolved/resolving at last follow-up. Of note, menstrual bleeding in patients with endometriosis was not interrupted during AMY109 treatment. Pharmacokinetic analysis showed that AMY109 had a long half-life (40 days) and exhibited linear pharmacokinetics across all cohorts.

**Conclusion:**

AMY109 demonstrated acceptable safety and pharmacokinetic profiles in HVs (single dose of 0.6–5.0 mg/kg) and patients with endometriosis (multiple doses of 0.8–5.0 mg/kg/month for 6 months). These results support further clinical development of AMY109 for endometriosis and other diseases influenced by interleukin-8.

**Trial registration number:**

AMY001JG study; JapicCTI-183841. Trial registration date: January 25, 2018. Date of first participant’s enrolment: February 26, 2018.

Endometriosis is a chronic, inflammatory, estrogen-dependent gynecologic disorder frequently associated with dysmenorrhea, nonmenstrual pelvic pain (NMPP), and infertility (1). Globally, endometriosis affects roughly 10% (190 million) of women of reproductive age (2, 3). The available treatment options include pain medications, hormonal treatments, or surgery (4, 5, 6). However, hormonal treatments often have adverse effects. For example, gonadotropin-releasing hormone agonists/antagonists can cause menopausal symptoms or decreased bone density, progestogens may lead to thrombin formation, and pain may recur after treatment ends (7, 8). Additionally, fertility can be impacted because hormonal treatments disrupt menstrual cycles and ovulation (7, 9). Although surgical treatments can effectively manage pain (10), a high rate of recurrence (22%–55%) has been reported (11), and symptoms can recur within 2–7 years of laparoscopic excision (12, 13, 14, 15, 16, 17). Consequently, there is an unmet need for noninvasive, nonhormonal, effective, and safe treatment options for patients with endometriosis.

To address this need, inflammation was investigated as a potential therapeutic target in endometriosis. Interleukin-8 (IL-8) has been identified as a key chemokine mediator of inflammation (18); elevated levels of IL-8 were found in the peritoneal fluid of patients with endometriosis when patients were grouped by treatment status or by the revised American Society for Reproductive Medicine staging criteria (19, 20, 21).

AMY109 was originally developed as a long-acting humanized anti–IL-8 recycling antibody to provide a more convenient treatment option than conventional antibody therapy, allowing for smaller doses and less frequent administration (22). In a preclinical animal model of endometriosis, AMY109 (2 and 10 mg/kg) significantly reduced the volume of nodular lesions and endometriosis-related adhesions, improved modified revised American Society for Reproductive Medicine scores, and induced atrophic changes in endometrial fibrosis. Studies assessing AMY109 safety in healthy animal models have reported that high doses of AMY109 (up to 200 mg/kg) produced no abnormal health measures (e.g., female menstrual cycles, body weight, electrocardiography, hematology, or urinalysis), except for reversible injection site reactions (22). Together, these data suggest that AMY109 represents a viable, nonhormonal treatment option for patients with endometriosis while maintaining a physiological menstrual cycle. Herein, we report the first phase 1 trial (AMY001JG) of AMY109 that assessed its safety, tolerability, and pharmacokinetic/pharmacodynamic profile in healthy volunteers (HVs) and in patients with endometriosis.

## Materials and methods

### Study design

**Figure 1:**
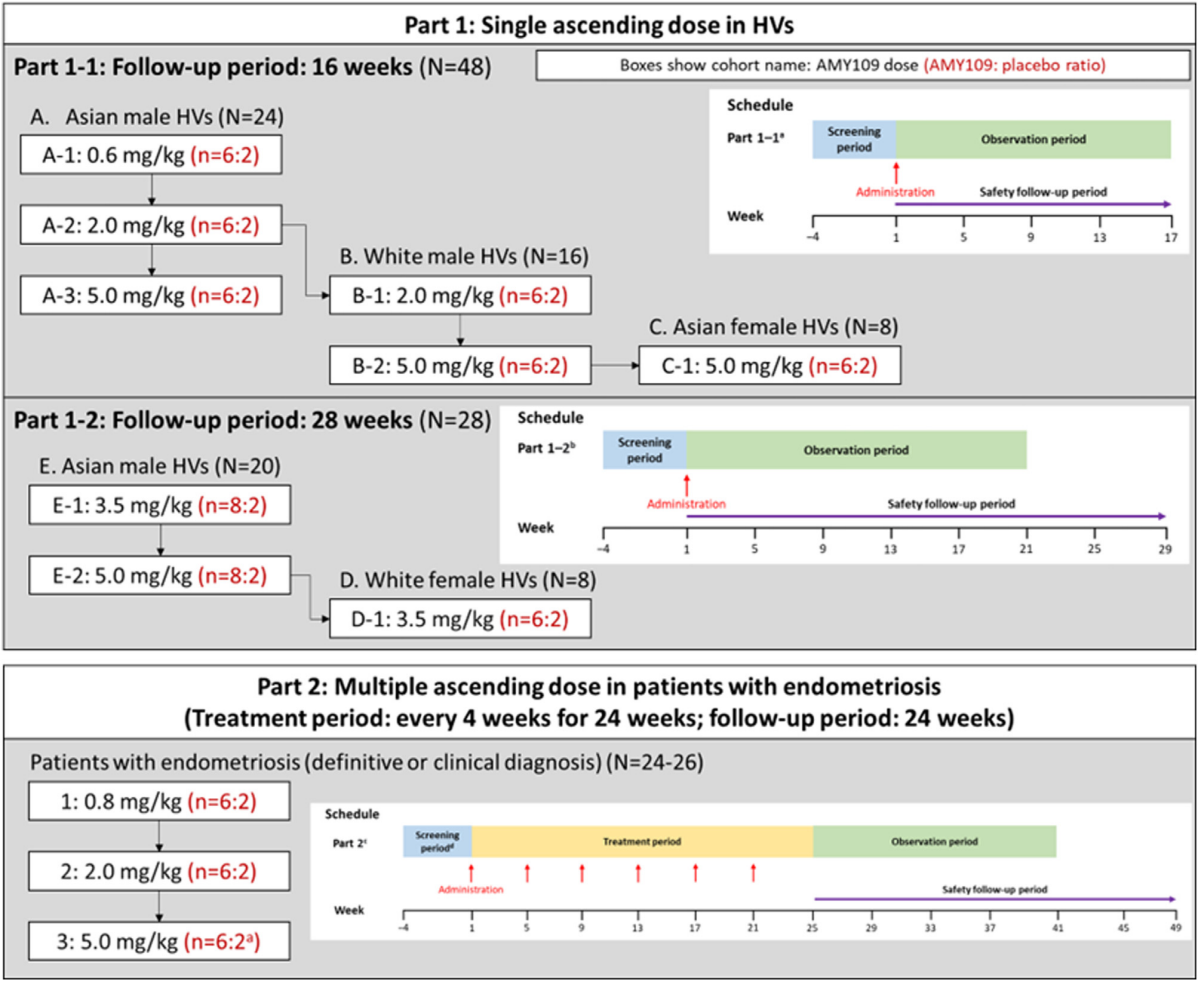
Planned number of patients and study schedule for each study cohort in parts 1-1, 1-2, and 2 of AMY001JG. Part 1 consisted of a single ascending dose with sequential opening of cohorts of healthy volunteers (HVs), and part 2 comprised the sequential opening of cohorts of Asian patients with endometriosis who received multiple ascending doses of AMY109 or placebo every 4 weeks over 24 weeks (6 doses in total). ^a^Up to 10 patients was able to be randomized to receive AMY109 or placebo in a 6:2 ratio for part 2 cohort 3. The actual enrolled numbers of patients were 7 and 3 for AMY109 and placebo, respectively. n = planned number of participants on the AMY109-to-placebo ratio.

AMY001JG was a 2-part, randomized, double-blind, placebo-controlled study of AMY109 in HVs (part 1) and patients with endometriosis (part 2) and was conducted at six sites in Japan and Taiwan (Fig. 1). The primary objective was to evaluate the safety and tolerability of AMY109 in both parts.

Part 1 was a single ascending-dose study, where sequentially opened, diverse cohorts of HVs were randomized to receive a single prespecified subcutaneous (SC) dose of AMY109 (0.6, 2.0, 3.5, or 5.0 mg/kg) or placebo. Part 1-1 (observation and follow-up period [FUP], 16 weeks) included Asian male HVs (cohorts A-1 to A-3), White male HVs (cohorts B-1 and B-2), and Asian female HVs of nonchildbearing potential (cohort C-1), all of whom had received doses up to 5.0 mg/kg of AMY109 or placebo. In part 1-2, the observation period was extended to 20 weeks, and the FUP was 28 weeks and comprised three cohorts (E-1, E-2, and D-1). Cohorts E-1 and E-2 were additional cohorts corresponding to A-2 and A-3, respectively, and received 3.5 mg/kg and 5.0 mg/kg AMY109, respectively, or placebo, for more stringent safety monitoring of upper respiratory tract infections (URTIs) and IL-8 levels, before to the initiation of part 2. Cohort D-1 enrolled White premenopausal female HVs and aimed primarily to determine the presence or absence of racial differences in pharmacokinetics. Because URTIs were observed in cohorts E-1 and E-2, the dose for cohort D-1 was reduced from the planned dose of 8.0 mg/kg to 3.5 mg/kg.

Part 2 was the multiple ascending-dose part of the study, where sequential cohorts of patients with endometriosis were randomized to receive once-monthly SC doses of 0.8 mg/kg (cohort 1), 2.0 mg/kg (cohort 2), and 5.0 mg/kg (cohort 3) AMY109 or placebo for 24 weeks. After administration of the final dose, safety follow-up occurred for a further 24 weeks. Further details on randomization, blinding, dose-escalation procedures, and statistical methods are included in the Supplemental Methods (available online).

The study was registered with the Japan Pharmaceutical Information Center (JapicCTI-183841).

### Study participants

For part 1, HVs were recruited if they met the following inclusion criteria: age of ≥20 to <55 years for males or ≥20 to <65 years for patients; body mass index of ≥18.5 to <25.0 kg/m^2^ for Asian HVs or ≥18.5 to <30.0 kg/m^2^ for White HVs; and no evidence of any active or chronic disease. Female HVs had to be surgically sterile, postmenopausal, or on a contraceptive regimen for ≥60 days before the screening period.

Part 2 included patients aged ≥20 to <50 years with a laparoscopic or clinical diagnosis of endometriosis (detected by a transvaginal or transanal ultrasound), who had ≥1 regular menstrual cycle (21–38 days) during the screening period and reported moderate-to-severe endometriosis-associated dysmenorrhea (score of ≥40 mm on the visual analogue scale [VAS] for ≥2 days of the screening period).

### Endpoints and outcomes

The primary endpoint was the proportion of participants experiencing adverse events (AEs). The safety population was defined as all participants who had received AMY109 or placebo. Safety assessments consisted of monitoring and recording AEs, including serious AEs (SAEs), AEs of special interest, and selected AEs, performing protocol-specified safety laboratory assessments, and measuring protocol-specified vital signs.

The secondary endpoint was the AMY109 plasma concentration, measured by Sandwich enzyme-linked immunosorbent assay using anti-AMY109 monoclonal antibody, which recognizes the fragment crystallizable region of AMY109 as capture reagent and IL-8 and anti-IL-8 monoclonal antibody as detector reagents. Pharmacokinetic parameters were stratified by treatment group and presented as peak plasma concentration (C_max_), time to C_max_ (T_max_), area under the curve (AUC) extrapolated to infinity (AUC_inf_) and to the last measurable concentration (AUC_last_), and elimination half-life (t_1/2_).

Exploratory endpoints included free IL-8 plasma levels (in parts 1 and 2) presented as the maximum free IL-8 level (E_max_) and the area under the time-free IL-8 concentration curve, preliminary efficacy on the basis of the VAS and modified Biberoglu and Behrman Scale (mBBS) pain scores via electronic patient-reported outcome methods and analgesic use (in part 2).

### Classification method for AEs

Adverse events reported in the study were coded according to the International Council for Harmonisation (ICH) Medical Dictionary for Regulatory Activities version 20.1 criteria. The events shown in the Results section and relevant tables are described using the event names coded by this method.

### Study ethics

The protocol of AMY001JG was reviewed and approved by each site’s Institutional Review Board or Ethics Committee. The study was conducted in accordance with the ICH of Technical Requirements for Registration of Pharmaceuticals for Human Use E6 guideline for Good Clinical Practice, the ICH E2A guidelines, the principles of the Declaration of Helsinki, and all laws and regulations of Japan and Taiwan. Data are reported according to the Consolidated Standards of Reporting Trials reporting guidelines (23). Written informed consent was obtained from all study participants.

### Statistical analysis

The planned sample size was eight participants per cohort in part 1 (AMY109-to-placebo ratio of 6:2), except for cohorts E-1 and E-2 (10 participants each, AMY109-to-placebo ratio of 8:2), and 8–10 participants in part 2 (AMY109-to-placebo ratio of 6:2). Target sample sizes were not based on power calculations.

Descriptive statistics (mean, median, minimum/maximum, percentage, and SD) were calculated for safety and pharmacokinetic parameters, free IL-8 plasma levels, and VAS/mBBS pain scores. Pharmacokinetic analysis software WinNonlin (Phoenix version 8.3) was used for parameter analysis. Estimated free IL-8 plasma levels were calculated using measured total IL-8 plasma levels and the dissociation constant of AMY109 (Supplemental Methods).

## Results

### Participant characteristics

This study was conducted between February 15, 2018, and July 5, 2022. In each cohort, participants were randomized to receive either AMY109 (n = 6–8) or placebo (n = 2–3; Fig. 1).

In part 1, 76 HVs (60 males and 16 females) were enrolled and randomized to either AMY109 (n = 58) or placebo (n = 18). Cohorts B-1, B-2, and D-1 included 24 White and 52 Asian HVs. During part 1, one HV from cohort B-1 withdrew study consent and subsequently discontinued the study.

During part 2, 26 Asian patients with endometriosis were enrolled and randomized to AMY109 (n = 19) or corresponding placebo (n = 7). Five patients withdrew during the treatment period, and one withdrew during the safety FUP, due to withdrawal of consent (n = 2) and SAE, physician decision, pregnancy, and loss to follow-up (n = 1 each).

Overall, 75 HVs and 20 patients with endometriosis completed the study (Supplemental Fig. 1, available online). All enrolled participants were included in the safety, pharmacokinetic, and other exploratory analyses. Baseline demographics of participants (parts 1 and 2) are summarized in Supplemental Tables 1 and 2 (available online).

### Primary endpoint: safety

#### Overall

Most AEs were grades 1–2 and had resolved or improved by the final follow-up. There was no correlation between AMY109 dose and AE occurrence (Supplemental Tables 3 and 4). Administration was discontinued in one patient with grade 3 invasive ductal breast carcinoma in part 2 (considered unrelated to AMY109 by the study investigator); however, there were no other AEs leading to discontinuation or death.

In HVs (part 1), events suggestive of URTI (defined by appropriate medical review) were frequently observed (Table 1 and Supplemental Table 5). In patients with endometriosis (part 2), vomiting, nasopharyngitis, diarrhea, nausea, and vaccination site pain were more common with AMY109 (Table 2). In both parts, there were no particular concerns about injection site reactions observed in nonclinical findings, and no AEs were thought to be attributed to antidrug antibody (ADA) expression.

**Table 1:** Summary of adverse events occurring between baseline and the end of the follow-up period in healthy volunteers administered a single subcutaneous dose of AMY109 of 0.6–5.0 mg/kg (part 1).

n (%)^a^	AMY109 dose, mg/kg					Placebo (n = 18)
	0.6 (n = 6)	2.0 (n = 12)	3.5 (n = 14)	5.0 (n = 26)	Total (n = 58)	
Any AE	3 (50.0)	1 (8.3)	6 (42.9)	13 (50.0)	23 (39.7)	9 (50.0)
Any SAE	0	0	0	1 (3.8)	1 (1.17)	0
Withdrawal due to AE, n (%)	0	0	0	0	0	0
Common AEs,^b^ n (%)						
Oropharyngeal pain	0	0	3 (21.4)	2 (7.7)	5 (8.6)	2 (11.1)
Pharyngitis	0	0	1 (7.1)	4 (15.4)	5 (8.6)	1 (5.6)
URTI	0	1 (8.3)	1 (7.1)	2 (7.7)	4 (6.9)	1 (5.6)
Upper respiratory tract inflammation	0	0	3 (21.4)	0	3 (5.2)	0

*Note:* AE = adverse event; SAE = serious adverse event; URTI = upper respiratory tract infection.

a Number of participants who experienced at least one AE.

b Occurring in ≥5% of participants.

**Table 2:** Summary of adverse events occurring between baseline and the end of the follow-up period in female patients with endometriosis administered a once-monthly subcutaneous dose of AMY109 of 0.8–5.0 mg/kg for 6 months (part 2).

n (%)^a^	AMY109 dose, mg/kg				Placebo (n = 7)
	0.8 (n = 6)	2.0 (n = 6)	5.0 (n = 7)	Total (n = 19)	
Any AE	6 (100.0)	3 (50.0)	4 (57.1)	13 (68.4)	3 (42.9)
Any SAE	1 (16.7)	1 (16.7)	0	2 (10.5)	0
Withdrawal due to AE, n (%)	1 (16.7)	0	0	1 (5.3)	0
Common AEs,^b^ n (%)					
Vomiting	3 (50.0)	2 (33.3)	0	5 (26.3)	0
Nasopharyngitis	2 (33.3)	1 (16.7)	1 (14.3)	4 (21.1)	0
Diarrhea	2 (33.3)	1 (16.7)	0	3 (15.8)	1 (14.3)
Nausea	2 (33.3)	1 (16.7)	0	3 (15.8)	0
Vaccination site pain	0	1 (16.7)	2 (28.6)	3 (15.8)	0

*Note:* AE = adverse event; SAE = serious adverse event.

a Number of participants who experienced at least one AE.

b Occurring in ≥15% of participants.

#### Single ascending dose in HVs (part 1)

Overall, 42.1% (n = 32/76) of HVs experienced an AE. Adverse events were observed in 39.7% (n = 23/58) in the AMY109 group and 50.0% (n = 9/18) in the placebo group (Table 1 and Supplemental Table 3). The most common AEs with AMY109 were oropharyngeal pain and pharyngitis (each 8.6%, n = 5/58), URTI (6.9%, n = 4/58), and upper respiratory tract inflammation (5.2%, n = 3/58; Table 1). Most AEs were mild to moderate in severity, unrelated to AMY109, and all had resolved by the last follow-up.

In part 1, events suggestive of URTI were the most common AEs (20.7% [n = 12/58] with AMY109 and 22.2% [n = 4/18] with placebo). One SAE of grade 3 epiglottitis, assessed as unrelated to AMY109 by the study investigator, was reported (Supplemental Table 5). This SAE, which occurred in part 1-1 (cohort A-3), prompted a temporary study halt and unblinding of all available cases. As a result, the limited number of subjects precluded definitive conclusions regarding infection associated with AMY109. Consequently, part 1-2 was conducted with the addition of two new cohorts (E-1 and E-2), dose reduction in D-1 (10.0 mg/kg to 8.0 mg/kg), and implementation of enhanced safety measures, including swab tests.

Results from cohorts E-1 and E-2 showed several events suggestive of URTI, although a causal relationship was not strongly evidenced. This led to a second study halt and unblinding of cohorts E-1 and E-2. The unblinding did not yield a clear conclusion on the relationship between AMY109 and AEs suggestive of a URTI, and swab tests revealed no clear microorganism trends or opportunistic infections. Nevertheless, a dose of 3.5 mg/kg, which was lower than the dose that had already been assessed to be tolerable, was selected for cohort D-1 to evaluate pharmacokinetic data in White premenopausal female HVs, and close monitoring was continued in subsequent cohorts.

Detected microorganisms that may have contributed to URTI events were viruses, including human coronavirus in six events (5 with AMY109 and 1 with placebo), human rhinovirus/enterovirus in two events (both with AMY109), other viruses in two events (both with AMY109), and various bacteria in four events (all with AMY109). Some of these microorganisms were also detected at screening. Events suggestive of infection (defined by appropriate medical review) were also reviewed, revealing no clear dose-dependent trend (Supplemental Table 6).

#### Multiple ascending dose in patients with endometriosis (part 2)

Overall, 61.5% (n = 16/26) of patients with endometriosis experienced an AE. Adverse events were observed in 68.4% (n = 13/19) in the AMY109 group and 42.9% (n = 3/7) in the placebo group (Table 2 and Supplemental Table 4). The most common AEs with AMY109 were vomiting (26.3%, n = 5/19), nasopharyngitis (21.1%, n = 4/19), diarrhea, nausea, and vaccination site pain (each 15.8%, n = 3/19; Table 2). There were no events suggestive of infection to be related to AMY109 considered by the study investigators (Supplemental Table 7).

Four patients with AMY109 experienced six grade ≥3 AEs (abdominal pain, invasive ductal breast carcinoma, syncope, cervical dysplasia, endometriosis [reported term, worsening of endometriosis], and mucinous breast carcinoma; each n = 1). Two patients experienced four SAEs; one patient reported invasive ductal breast carcinoma, and the other reported one SAE each of cervical dysplasia, endometriosis, and mucinous breast carcinoma. All grade ≥3 AEs and SAEs were deemed unrelated to AMY109 by study investigators. At the last follow-up, the patient with mucinous breast carcinoma was recovering; all other SAEs had resolved.

In part 2, events suggestive of URTIs were observed in 21.1% of patients (n = 4/19) in the AMY109 group and 14.3% (n = 1/7) in the placebo group (Supplemental Table 8). No clear relationship was established between the occurrence of upper respiratory tract–related AEs or AE severity and AMY109 dosage.

Gastrointestinal-related events were more frequently observed with AMY109 (31.6%, n = 6/19) than with placebo (14.3%, n = 1/7); however, no dose-dependent trend in occurrence was observed for AMY109 (0.8 mg/kg, 66.7%; 2 mg/kg, 33.3%; 5 mg/kg, 0; Supplemental Table 4). All gastrointestinal-related events were judged to be unrelated to AMY109 by study investigators, and factors that were reported as possible causes for these events included the effects of endometriosis or menstruation, concomitant medications (e.g., analgesics), or other factors.

No menstrual-related AEs (e.g., abnormal bleeding or irregular menstruation) were reported.

In part 2 cohort 2, one patient experienced pregnancy after receiving two doses of AMY109 and discontinued the administration. The birth outcome was reported as normal, and the patient and infant were healthy.

### Secondary endpoint: pharmacokinetics

All pharmacokinetic parameters are summarized in Supplemental Table 9. Across parts 1 and 2, AMY109 C_max_, AUC_inf_, and AUC_last_ increased in a dose-dependent manner. The median T_max_ (7.0–13.4 days) and mean t_1/2_ (39.0–55.8 days) remained similar across all cohorts and were not dose dependent.

**Figure 2:**
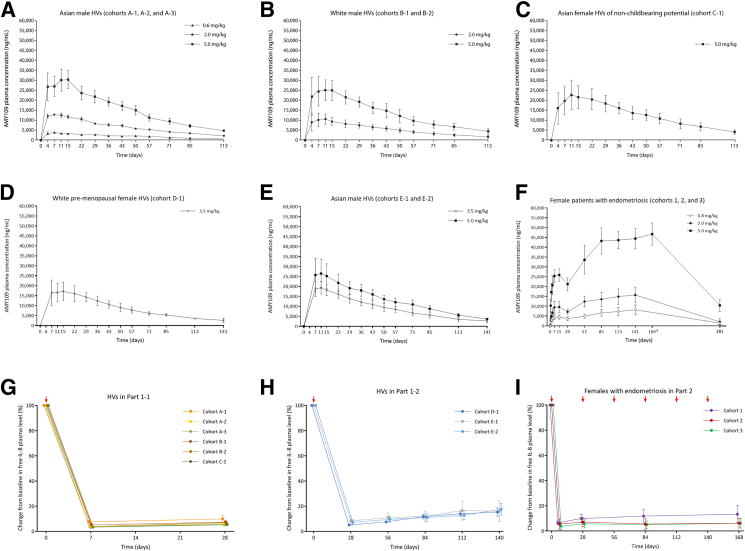
Pharmacokinetics of AMY109 and free interleukin-8 (IL-8) plasma levels in parts 1 and 2. Mean ± SD of AMY109 plasma concentrations over time after single-dose subcutaneous administration of AMY109 in Asian male healthy volunteers (HVs) (**A**); White male HVs (**B**); Asian female HVs of nonchildbearing potential (**C**); White premenopausal female HVs (**D**); Asian male HVs (cohorts E-1 and E-2) (**E**); and AMY109 plasma concentrations in the multiple ascending-dose part in patients with endometriosis in part 2 (**F**). Mean ± SD percent change in free IL-8 plasma levels from baseline to end of follow-up period in HVs in part 1-1 (**G**), HVs in part 1-2 (**H**), and patients with endometriosis in part 2 (**I**). Red arrows indicate the timepoints at which AMY109 or placebo was administered. ^a^At day 169, mean (SD) AMY109 plasma concentrations were not calculated in cohort 1 (0.8 mg/kg) or 2 (2.0 mg/kg) due to limited sample size (n = 2 in each), caused by sampling window deviation and early study termination.

AMY109 exhibited linear pharmacokinetics in Asian and White male HVs (Fig. 2A [cohorts A-1, A-2, and A-3], 2B [cohorts B-1 and B-2], and 2E [cohorts E-1 and E-2]), healthy Asian females of nonchildbearing potential (Fig. 2C [cohort C-1]), White premenopausal female HVs (Fig. 2D [cohort D-1]), and patients with endometriosis (Fig. 2F [cohorts 1–3]). No major differences in pharmacokinetics were observed between Asian and White HVs (Supplemental Table 10), males and females, or HVs and patients with endometriosis (Supplemental Table 9).

Although 10.3% (n = 6/58) of HVs in part 1 and 26.3% (n = 5/19) of patients with endometriosis in part 2 in the AMY109 group were ADA-positive, the presence of ADAs did not affect the pharmacokinetic profile of AMY109.

### Exploratory endpoint: pharmacodynamics

The estimated mean free IL-8 plasma levels (calculated as described in the Supplemental Methods) decreased rapidly after the first AMY109 dose in all cohorts. In part 1-1, the free IL-8 plasma levels remained low at day 28 (Fig. 2G) and from days 28 to 140 (Fig. 2H) after single-dose administration of AMY109. In part 2, low free IL-8 plasma levels were sustained with multiple-dose AMY109 administration in patients with endometriosis (Fig. 2I).

### Exploratory endpoint: relationship between safety and pharmacokinetics/pharmacodynamics

In correlation analyses of the relationship between safety and pharmacokinetics/pharmacodynamics, the probability of AEs and C_max_/AUC (Supplemental Fig. 2A–D) and of AEs and E_max_/area under the time-free IL-8 concentration curve (Supplemental Fig. 2E–H) showed no significant correlation.

### Exploratory endpoint: efficacy

In part 2, the mean VAS scores for dysmenorrhea decreased from baseline to the end of treatment (i.e., after dose 6) by 6.6, 18.0, and 16.9 mm in patients with endometriosis receiving AMY109 0.8, 2.0, and 5.0 mg/kg, respectively (Supplemental Table 11). The mean VAS scores for NMPP decreased by 0.4, 2.0, and 10.0 mm in the respective cohorts. No clear differences were observed in pain scores between the AMY109 and placebo groups. Although the mean mBBS scores for dysmenorrhea and NMPP and the number of days that patients required analgesic medication had decreased with AMY109 across all part 2 cohorts, these changes were not numerically different from those observed with placebo (Supplemental Table 12 and Supplemental Fig. 3).

## Discussion

The AMY001JG study is the first phase 1 trial to evaluate the safety of an anti-IL-8 recycling antibody and investigate the effects of pharmacologic IL-8 inhibition in patients with endometriosis. The primary endpoint was achieved, and AMY109 (0.6–5.0 mg/kg) was generally well tolerated when administered to HVs in a single SC dose and to females with endometriosis in multiple SC doses for up to 6 months. The expected biologically active AMY109 exposure at each dose level was determined by in vitro pharmacologic modeling and preclinical in vivo pharmacologic studies (data on file).

AMY109 was generally well tolerated, with no clear relationship observed between dosage and the proportion of participants experiencing AEs. Most of the commonly reported gastrointestinal-related events in part 2 (vomiting, diarrhea, and nausea) linked to patient backgrounds and concomitant medications.

Considering that IL-8 is an established neutrophil chemotactic factor (24, 25), individuals who receive AMY109 may have lower IL-8 levels and possibly an increased susceptibility to infection compared with the general population. However, there are no known reports that other IL-8 inhibitors are associated with infection. One grade 3 epiglottitis and several grade 1/2 events suggestive of URTIs were observed in part 1, leading to unblinding and the subsequent cohort addition of cohorts E-1 and E-2, lower dose adaptation in cohort D-1, and additional safety measures. Further investigation of the HVs or patients with events suggestive of URTIs found several viruses and bacteria; however, there was no clear trend among them or evidence of opportunistic infections. On the basis of the mechanism of action of AMY109, it was expected that most infection-related events would be of bacterial origin; however, these results did not confirm this expectation. Evaluation of URTI-related AEs (using the Bradford Hill criteria) did not suggest strong evidence for a causal relationship with AMY109. Despite appropriate use of swabs and bacterial cultures, a definitive diagnosis of infection was difficult, and the study was not powered to draw clear conclusions due to its small population size and the observed infection-related events that may occur in the general population. Therefore, it is difficult to determine the relationship between AMY109 and infections solely on the basis of the results of this study. However, the results obtained after single/multiple SC doses of AMY109 (up to 5 mg/kg) in HVs and patients with endometriosis did not indicate any apparent clinically significant concerns of URTI-related AEs. Further clinical development is supported, and future studies should continue to monitor for infectious events, including URTI-related AEs.

AMY109 displayed linear pharmacokinetics in HVs and patients with endometriosis, with dose-dependent increases in C_max_, AUC_inf_, and AUC_last_ observed across the 0.6–5.0 mg/kg dose range. Additionally, no major differences in pharmacokinetics were observed between the Asian and White HVs, suggesting that no race-specific dose modification is required in future global clinical trials of AMY109.

As expected, AMY109 showed a prolonged half-life in part 1 (mean, 39.0–55.8 days) and in preclinical studies (approximately 20 days) when compared with conventional antibodies that have not been engineered by the recycling antibody and ACT-Ig engineering technology (22). Drugs with longer half-lives allow for once-monthly dosing regimens, which are associated with higher patient convenience and adherence (26). Such findings have been similarly reported in the fields of hematology and oncology, whereby antibody candidates engineered with sequential monoclonal antibody recycling and recycling antibody technology, respectively, were associated with prolonged half-lives and a decreased need for frequent dosing (27, 28). Of note, although the half-life of AMY109 could not be measured in part 2 due to sampling timepoint limitations, it is expected to be similar to that found in part 1 on the basis of the assessment of day 29 pharmacokinetic parameters in patients with endometriosis.

AMY109 suppressed free IL-8 plasma levels; these low chemokine levels were sustained with multiple-dose administration. Several studies have discussed the role of IL-8 in endometriosis pathogenesis, particularly its ability to support endometrial cell adhesion, invasion, and implantation within the peritoneum and promote cell growth via autocrine signaling (18, 21, 29). Recently, it has been reported that infertile women with dysmenorrhea or pelvic pain had high IL-8 levels in peritoneal fluid that showed correlation with pain intensity (30). Taken together with increased IL-8 receptor expression in the eutopic endometrium of patients with endometriosis (18), the lowering of free IL-8 levels may prevent endometrial cell invasion, avert excessive cellular growth in patients with endometriosis, and alleviate pain. The 5-mg/kg once-monthly regimen provided maximum drug exposure and was well tolerated, which suggests that higher doses could potentially be tolerated. Importantly, there was no correlation between the probability of AEs and the estimated free IL-8 levels. Although the exploratory efficacy analyses showed decreased VAS and mBBS scores for dysmenorrhea and NMPP with AMY109 across all cohorts of patients with endometriosis, these scores were not numerically different from those observed with placebo.

The findings of this study, particularly regarding the efficacy of AMY109, should be interpreted cautiously due to several limitations. First, the sample size was relatively small across all cohorts, limiting conclusions regarding efficacy. Second, the treatment period may have been too short to accurately assess improvements in endometrial lesions, given that AMY109 previously showed significant effects after 12 months of administration in preclinical studies (22); future studies should include a larger sample size and have extended treatment and FUPs. Third, there were no strict inclusion criteria for pain assessment (i.e., the population likely had high interperson variation and low baseline VAS scores). Lastly, because the IL-8 levels were only examined in plasma and given that IL-8 receptors are highly expressed in endometriotic tissue in patients with endometriosis (31), the optimal dose and effects of AMY109 should be evaluated in endometriotic tissue in future studies.

## Conclusion

The first-in-human, phase 1 trial demonstrated that AMY109 has acceptable safety and good pharmacokinetic/pharmacodynamic profiles with rapid and long-term suppression of free IL-8 plasma levels. AMY109 was well tolerated in HVs (single-dose administration up to 5 mg/kg) and in patients with endometriosis (multiple-dose administration up to 5 mg/kg/month once-monthly for 6 months), supporting its further evaluation for the treatment of endometriosis. Of note, AEs suggestive of URTIs were observed; AEs (including those suggestive of infections) and AMY109 efficacy are being monitored in further studies including the ongoing phase 2 AMY106EU study (ISCTRN15654320).

## Declaration of Interests

P.-H.W. reports funding from Chugai Pharmaceutical Co., Ltd., for the submitted work. S.-M.H. reports funding from Chugai Pharmaceutical Co., Ltd., for the submitted work. S.M. reports funding from Chugai Pharmaceutical Co., Ltd., for the submitted work. R.H. reports funding from Chugai Pharmaceutical Co., Ltd., for the submitted work. C.-A.C. reports employee, stock options, travel support, and nonvoting support member of dose escalation committee for Chugai Pharmaceutical Co., Ltd. A.N.-K. reports travel support, inventor of the patents of anti–interleukin-8 antibodies and endometriosis (all rights have been assigned to Chugai), nonvoting support member of dose escalation committee etc., stock and employee of Chugai Pharmaceutical Co., Ltd. M.S. reports employee, stock options, travel support, and nonvoting support member of dose escalation committee for Chugai Pharmaceutical Co., Ltd. K.N. is an employee of Chugai Pharmaceuticals Co., Ltd. J.M. is an employee of Chugai Pharmaceuticals Co., Ltd. M.S. reports funding from Chugai Pharmaceuticals Co., Ltd., for the submitted work.
