# Supplementary material for: Safety and pharmacology of AMY109, a long-acting anti–interleukin-8 antibody, for endometriosis: a double-blind, randomized phase 1 trial
Source: F S Rep. 2025 May 2;6(3):261–9. doi: 10.1016/j.xfre.2025.04.009 (PMC12496429; doi:10.1016/j.xfre.2025.04.009)

**Safety and pharmacology of AMY109, a long-acting anti-interleukin-8 antibody, for endometriosis: a double-blind, randomized phase 1 trial**

Peng-Hui Wang, MD, PhD^a^, Sheng-Mou Hsiao, MD^b^, Shunji Matsuki, MD, PhD^c^, Ryuzo Hanada, MD, PhD^d^, Chun-An Chen, MSc^e^, Ayako Nishimoto-Kakiuchi, PhD^e^, Mayuko Sekiya, DVM^f^, Kiyohiko Nakai, MSc^e^, Junnosuke Matsushima, MSE^g^, Mari Sawada, MD^h^

^a^ Department of Obstetrics and Gynecology, Taipei Veterans General Hospital and National Yang Ming Chiao Tung University, Taipei 11217, Taiwan.
^b^ Department of Obstetrics and Gynecology, Far Eastern Memorial Hospital, New Taipei 220216, Taiwan.

^c^ Department of Clinical Research Center, Souseikai Fukuoka Mirai Hospital, 3-5-1, Kashiiteriha, Higashi-ku, Fukuoka, 813-0017, Japan

^d^ Souseikai Sumida Hospital, 1-29-1, Honjo, Sumida-ku, Tokyo, 130-0004, Japan.
^e^ Translational Research Division, Chugai Pharmaceutical Co., Ltd., 2-1-1 Nihonbashi-Muromachi Chuo-ku, Tokyo 103-8324, Japan.
^f^ Drug Safety Division, Chugai Pharmaceutical Co., Ltd., 2-1-1 Nihonbashi-Muromachi Chuo-ku, Tokyo 103-8324, Japan.

^g^ Clinical Development Division, Chugai Pharmaceutical Co., Ltd., 2-1-1 Nihonbashi-Muromachi Chuo-ku, Tokyo 103-8324, Japan.
^h^ Department of Obstetrics and Gynecology, Kurashiki Medical Center, Okayama 710-0826, Japan.

**Corresponding Author:** Peng-Hui Wang

Email: phwang@vghtpe.gov.tw; pongpongwang@gmail.com

ORCiD: 0000-0002-6048-8541

# Supplemental Material

Contents

[Supplemental Material 2](#_Toc195867235)

[Supplemental Methods 3](#_Toc195867236)

[Safety Monitoring and Cohort Transition 3](#_Toc195867237)

[Rationale for dose setting in Part 2 3](#_Toc195867238)

[Randomization and Blinding 4](#_Toc195867239)

[Assessment of Pain (Exploratory Endpoint) 4](#_Toc195867240)

[Free Interleukin-8 Plasma Levels 4](#_Toc195867241)

[Supplemental Tables 5](#_Toc195867242)

[Supplemental Table 1 5](#_Toc195867243)

[Supplemental Table 2 6](#_Toc195867244)

[Supplemental Table 3 7](#_Toc195867245)

[Supplemental Table 4 9](#_Toc195867246)

[Supplemental Table 5 11](#_Toc195867247)

[Supplemental Table 6 12](#_Toc195867248)

[Supplemental Table 7 13](#_Toc195867249)

[Supplemental Table 8 14](#_Toc195867250)

[Supplemental Table 9 15](#_Toc195867251)

[Supplemental Table 10 16](#_Toc195867252)

[Supplemental Table 11 17](#_Toc195867253)

[Supplemental Table 12 18](#_Toc195867254)

[Supplemental Figures 19](#_Toc195867255)

[Supplemental Fig. 1. 19](#_Toc195867256)

[Supplemental Fig. 2. 20](#_Toc195867257)

[Supplemental Fig. 3. 21](#_Toc195867258)

# Supplemental Methods

## Safety Monitoring and Cohort Transition

Safety was monitored in each dose cohort in Parts 1 and 2. Plasma and urine samples were collected for safety, pharmacokinetic, and immunogenicity analyses 1 day prior to AMY109 administration and at varying time points post-administration for different cohorts. Progression to the next cohort occurred only after blinded assessment of safety and tolerability of the previous cohort by the safety monitoring committee (which included the study investigators and the Sponsor’s medical representative). Following confirmation of safety/tolerability of previous doses of AMY109 in healthy volunteers (HVs) in Part 1, dose escalations were implemented. The safety evaluation in the highest dose cohort of patients (i.e., Part 2 Cohort 3) was also performed in the same manner, although not as the cohort transition. Finally, the safety and tolerability up to Part 2 Cohort 3 were reconfirmed based on the absence of dose-dependent increase in the incidence of AMY109-related AEs, and the absence of unacceptable AEs in patients with endometriosis after unblinding.

The screening period for patients with endometriosis in Part 2 could be extended by 10 days to ensure inclusion of the period of uterine bleeding or to adjust the visit schedule for patient convenience.

## Rationale for dose setting in Part 2

The doses in Part 2 are selected based on exposures experienced in Part 1 and other phase 1 study of AMY109 conducted in patients with solid tumors (Japan Registry of Clinical Trials ID: jRCT2080225101). The exposure of multiple doses of 0.8 mg/kg every 4 weeks in the first cohort corresponds to the exposure at the single dose of 2 mg/kg.

## Randomization and Blinding

The randomization list was generated by an independent vendor for both study Parts. No stratification factors were set at randomization. To ensure confidentiality, the blinded randomization list was not available to the study team.

Randomization of study treatment was performed by unblinded pharmacists at the study sites according to pre-specified procedures in Part 1 and via an interactive voice or web-based response system in Part 2. The formulation of AMY109 or placebo were identical in size, shape, color, and smell, and the packaging and labelling were designed to maintain blinding of both site staff and study participants.

## Assessment of Pain (Exploratory Endpoint)

In Part 2, mean visual analog scale (VAS) and modified Biberoglu and Behrman Scale (mBBS) pain scores at each dose were recorded from the day of drug administration until the day before the subsequent dose. For example, pain scores for dose 1 were a mean of daily reported pain scores from the day of administration for dose 1 until the day prior to dose 2 administration. Pain scores for dysmenorrhea were only included on days with uterine bleeding, while non-menstrual pelvic pain (NMPP) scores excluded days with uterine bleeding.

## Free Interleukin-8 Plasma Levels

Free interleukin (IL)-8 plasma levels were estimated using the measured total IL-8 plasma levels and the dissociation constant (K_d_) of AMY109 as follows:

1. Free IL-8 plasma level = total IL-8 plasma level – AMY109:IL-8 complex plasma concentration
2. AMY109:IL-8 complex plasma concentration = (total IL-8 plasma level + AMY109 plasma concentration + K_d_ – [(total IL-8 plasma level + AMY109 plasma concentration + K_d_)^2^ – 4 × total IL-8 plasma level × AMY109 plasma concentration]^0.5^) × 0.5

# Supplemental Tables

## Supplemental Table 1

Baseline participant demographics of healthy volunteers administered a single subcutaneous dose of AMY109 0.6–5.0 mg/kg (Part 1).

| **Characteristic** | **AMY109 dose, mg/kg** | | | | | **Placebo (n=18)** |
| --- | --- | --- | --- | --- | --- | --- |
|  | **0.6 (n=6)** | **2.0 (n=12)** | **3.5 (n=14)** | **5.0 (n=26)** | **Total (n=58)** |  |
| Age, mean (SD), years | 22.5 (2.1) | 30.7 (8.2) | 30.1 (9.4) | 39.2 (12.8) | 33.5 (11.7) | 31.3 (12.6) |
| Male, n (%) | 6 (100.0) | 12 (100.0) | 8 (57.1) | 20 (76.9) | 46 (79.3) | 14 (77.8) |
| Female, n (%) | 0 | 0 | 6 (42.9) | 6 (23.1) | 12 (20.7) | 4 (22.2) |
| Asian, n (%) | 6 (100.0) | 6 (50.0) | 8 (57.1) | 20 (76.9) | 40 (69.0) | 12 (66.7) |
| White, n (%) | 0 | 6 (50.0) | 6 (42.9) | 6 (23.1) | 18 (31.0) | 6 (33.3) |
| Weight, mean (SD), kg | 58.5 (6.3) | 67.7 (7.0) | 64.8 (6.2) | 63.9 (11.5) | 64.4 (9.3) | 62.7 (9.0) |
| Height, mean (SD), cm | 170.9 (8.0) | 174.5 (2.9) | 169.3 (7.7) | 168.3 (10.0) | 170.1 (8.4) | 169.0 (8.0) |
| BMI, mean (SD), kg/m^2^ | 20.0 (1.3) | 22.2 (2.0) | 22.7 (2.5) | 22.4 (2.2) | 22.2 (2.2) | 21.9 (1.8) |

BMI = body mass index; SD = standard deviation.

## Supplemental Table 2

Baseline participant demographics of female patients with endometriosis administered once-monthly subcutaneous doses of AMY109 0.8–5.0 mg/kg for 6 months (Part 2).

| **Characteristic** | **AMY109 dose, mg/kg** | | | | **Placebo (n=7)** |
| --- | --- | --- | --- | --- | --- |
|  | **0.8 (n=6)** | **2.0 (n=6)** | **5.0 (n=7)** | **Total (n=19)** |  |
| Age, mean (SD), years | 38.3 (6.0) | 39.7 (6.3) | 40.1 (6.2) | 39.4 (5.8) | 41.9 (2.2) |
| Male, n (%) | 0 | 0 | 0 | 0 | 0 |
| Female, n (%) | 6 (100.0) | 6 (100.0) | 7 (100.0) | 19 (100.0) | 7 (100.0) |
| Asian, n (%) | 6 (100.0) | 6 (100.0) | 7 (100.0) | 19 (100.0) | 7 (100.0) |
| White, n (%) | 0 | 0 | 0 | 0 | 0 |
| Weight, mean (SD), kg | 54.4 (6.9) | 56.1 (5.9) | 55.3 (7.0) | 55.2 (6.3) | 64.0 (15.0) |
| Height, mean (SD), cm | 154.9 (4.9) | 160.8 (5.4) | 166.0 (5.4) | 160.9 (6.8) | 158.3 (6.8) |
| BMI, mean (SD), kg/m^2^ | 22.8 (3.8) | 21.6 (1.1) | 20.1 (2.7) | 21.4 (2.8) | 25.3 (4.1) |

BMI = body mass index; SD = standard deviation.

## Supplemental Table 3

Adverse events occurring between baseline and the end of the follow-up period in healthy volunteers who were administered a single subcutaneous dose of AMY109 0.6–5.0 mg/kg (Part 1).

| **n (%)^a^** | **AMY109 dose, mg/kg** | | | | | **Placebo (n=18)** |
| --- | --- | --- | --- | --- | --- | --- |
|  | **0.6 (n=6)** | **2.0 (n=12)** | **3.5 (n=14)** | **5.0 (n=26)** | **Total (n=58)** |  |
| Any AE | 3 (50.0) | 1 (8.3) | 6 (42.9) | 13 (50.0) | 23 (39.7) | 9 (50.0) |
| Infections and infestations | 1 (16.7) | 1 (8.3) | 3 (21.4) | 6 (23.1) | 11 (19.0) | 5 (27.8) |
| Pharyngitis | 0 | 0 | 1 (7.1) | 4 (15.4) | 5 (8.6) | 1 (5.6) |
| URTI | 0 | 1 (8.3) | 1 (7.1) | 2 (7.7) | 4 (6.9) | 1 (5.6) |
| Tonsillitis | 0 | 0 | 1 (7.1) | 1 (3.8) | 2 (3.4) | 0 |
| Epiglottitis | 0 | 0 | 0 | 1 (3.8) | 1 (1.7) | 0 |
| Gastroenteritis | 0 | 0 | 1 (7.1) | 0 | 1 (1.7) | 0 |
| Herpes zoster | 0 | 0 | 0 | 1 (3.8) | 1 (1.7) | 0 |
| Laryngitis | 0 | 0 | 0 | 1 (3.8) | 1 (1.7) | 0 |
| Rhinitis | 0 | 0 | 1 (7.1) | 0 | 1 (1.7) | 0 |
| Tinea infection | 1 (16.7) | 0 | 0 | 0 | 1 (1.7) | 2 (11.1) |
| Nasopharyngitis | 0 | 0 | 0 | 0 | 0 | 1 (5.6) |
| Sinusitis | 0 | 0 | 0 | 0 | 0 | 1 (5.6) |
| Respiratory, thoracic, and mediastinal disorders | 0 | 0 | 5 (35.7) | 3 (11.5) | 8 (13.8) | 4 (22.2) |
| Oropharyngeal pain | 0 | 0 | 3 (21.4) | 2 (7.7) | 5 (8.6) | 2 (11.1) |
| Upper respiratory tract inflammation | 0 | 0 | 3 (21.4) | 0 | 3 (5.2) | 0 |
| Rhinorrhea | 0 | 0 | 1 (7.1) | 1 (3.8) | 2 (3.4) | 2 (11.1) |
| Productive cough | 0 | 0 | 1 (7.1) | 0 | 1 (1.7) | 0 |
| Rhinitis allergic | 0 | 0 | 0 | 0 | 0 | 1 (5.6) |
| Vocal cord inflammation | 0 | 0 | 0 | 0 | 0 | 1 (5.6) |
| Gastrointestinal disorders | 0 | 0 | 2 (14.3) | 4 (15.4) | 6 (10.3) | 1 (5.6) |
| Nausea | 0 | 0 | 0 | 2 (7.7) | 2 (3.4) | 0 |
| Aphthous ulcer | 0 | 0 | 0 | 1 (3.8) | 1 (1.7) | 0 |
| Dental caries | 0 | 0 | 1 (7.1) | 0 | 1 (1.7) | 1 (5.6) |
| Enterocolitis | 0 | 0 | 1 (7.1) | 0 | 1 (1.7) | 0 |
| Stomatitis | 0 | 0 | 0 | 1 (3.8) | 1 (1.7) | 0 |
| Vomiting | 0 | 0 | 0 | 1 (3.8) | 1 (1.7) | 0 |
| Skin and subcutaneous tissue disorders | 0 | 0 | 2 (14.3) | 3 (11.5) | 5 (8.6) | 4 (22.2) |
| Urticaria | 0 | 0 | 1 (7.1) | 1 (3.8) | 2 (3.4) | 0 |
| Alopecia | 0 | 0 | 1 (7.1) | 0 | 1 (1.7) | 0 |
| Dermatitis contact | 0 | 0 | 0 | 1 (3.8) | 1 (1.7) | 2 (11.1) |
| Rash | 0 | 0 | 0 | 1 (3.8) | 1 (1.7) | 0 |
| Acne | 0 | 0 | 0 | 0 | 0 | 1 (5.6) |
| Dyshidrotic eczema | 0 | 0 | 0 | 0 | 0 | 1 (5.6) |
| Investigations | 1 (16.7) | 0 | 1 (7.1) | 1 (3.8) | 3 (5.2) | 1 (5.6) |
| Blood bilirubin increased | 0 | 0 | 1 (7.1) | 1 (3.8) | 2 (3.4) | 0 |
| Blood creatine phosphokinase increased | 1 (16.7) | 0 | 0 | 0 | 1 (1.7) | 1 (5.6) |
| Injury, poisoning and procedural complications | 1 (16.7) | 0 | 0 | 0 | 1 (1.7) | 1 (5.6) |
| Foreign body in eye | 1 (16.7) | 0 | 0 | 0 | 1 (1.7) | 0 |
| Heat illness | 0 | 0 | 0 | 0 | 0 | 1 (5.6) |
| Musculoskeletal and connective tissue disorders | 0 | 0 | 1 (7.1) | 0 | 1 (1.7) | 0 |
| Back pain | 0 | 0 | 1 (7.1) | 0 | 1 (1.7) | 0 |
| General disorders and administration site conditions | 0 | 0 | 0 | 0 | 0 | 2 (11.1) |
| Injection site pain | 0 | 0 | 0 | 0 | 0 | 1 (5.6) |
| Injection site reaction | 0 | 0 | 0 | 0 | 0 | 1 (5.6) |
| Immune system disorders | 0 | 0 | 0 | 0 | 0 | 1 (5.6) |
| Seasonal allergy | 0 | 0 | 0 | 0 | 0 | 1 (5.6) |
| Nervous system disorders | 0 | 0 | 0 | 0 | 0 | 1 (5.6) |
| Headache | 0 | 0 | 0 | 0 | 0 | 1 (5.6) |

^a^Number of participants who experienced at least one AE.
AE = adverse event; URTI = upper respiratory tract infection.

## Supplemental Table 4

Adverse events occurring between baseline and the end of the follow-up period in patients with endometriosis who were administered once-monthly subcutaneous doses of AMY109 0.8–5.0 mg/kg for 6 months (Part 2).

| **n (%)^a^** | **AMY109 dose, mg/kg** | | | | **Placebo (n=7)** |
| --- | --- | --- | --- | --- | --- |
|  | **0.8 (n=6)** | **2.0 (n=6)** | **5.0 (n=7)** | **Total (n=19)** |  |
| Any AE | 6 (100.0) | 3 (50.0) | 4 (57.1) | 13 (68.4) | 3 (42.9) |
| General disorders and administration site conditions | 4 (66.7) | 1 (16.7) | 3 (42.9) | 8 (42.1) | 1 (14.3) |
| Vaccination site pain | 0 | 1 (16.7) | 2 (28.6) | 3 (15.8) | 0 |
| Malaise | 2 (33.3) | 0 | 0 | 2 (10.5) | 0 |
| Infusion site erythema | 0 | 0 | 1 (14.3) | 1 (5.3) | 0 |
| Oedema | 1 (16.7) | 0 | 0 | 1 (5.3) | 0 |
| Pyrexia | 1 (16.7) | 0 | 0 | 1 (5.3) | 1 (14.3) |
| Gastrointestinal disorders | 4 (66.7) | 2 (33.3) | 0 | 6 (31.6) | 1 (14.3) |
| Vomiting | 3 (50.0) | 2 (33.3) | 0 | 5 (26.3) | 0 |
| Diarrhoea | 2 (33.3) | 1 (16.7) | 0 | 3 (15.8) | 1 (14.3) |
| Nausea | 2 (33.3) | 1 (16.7) | 0 | 3 (15.8) | 0 |
| Abdominal pain | 2 (33.3) | 0 | 0 | 2 (10.5) | 0 |
| Oral pain | 1 (16.7) | 0 | 0 | 1 (5.3) | 0 |
| Stomatitis | 1 (16.7) | 0 | 0 | 1 (5.3) | 0 |
| Respiratory, thoracic, and mediastinal disorders | 5 (83.3) | 0 | 0 | 5 (26.3) | 1 (14.3) |
| Oropharyngeal pain | 2 (33.3) | 0 | 0 | 2 (10.5) | 1 (14.3) |
| Oropharyngeal discomfort | 1 (16.7) | 0 | 0 | 1 (5.3) | 1 (14.3) |
| Rhinorrhea | 1 (16.7) | 0 | 0 | 1 (5.3) | 0 |
| Sneezing | 1 (16.7) | 0 | 0 | 1 (5.3) | 0 |
| Skin and subcutaneous tissue disorders | 3 (50.0) | 1 (16.7) | 1 (14.3) | 5 (26.3) | 1 (14.3) |
| Dermatitis | 1 (16.7) | 0 | 1 (14.3) | 2 (10.5) | 1 (14.3) |
| Dermatitis contact | 1 (16.7) | 1 (16.7) | 0 | 2 (10.5) | 0 |
| Eczema | 1 (16.7) | 0 | 0 | 1 (5.3) | 0 |
| Rash | 1 (16.7) | 0 | 0 | 1 (5.3) | 0 |
| Telangiectasia | 1 (16.7) | 0 | 0 | 1 (5.3) | 0 |
| Infections and infestations | 2 (33.3) | 1 (16.7) | 1 (14.3) | 4 (21.1) | 1 (14.3) |
| Nasopharyngitis | 2 (33.3) | 1 (16.7) | 1 (14.3) | 4 (21.1) | 0 |
| Conjunctivitis | 1 (16.7) | 0 | 0 | 1 (5.3) | 0 |
| URTI | 0 | 0 | 0 | 0 | 1 (14.3) |
| Nervous system disorders | 2 (33.3) | 2 (33.3) | 0 | 4 (21.1) | 0 |
| Dizziness | 2 (33.3) | 0 | 0 | 2 (10.5) | 0 |
| Headache | 0 | 1 (16.7) | 0 | 1 (5.3) | 0 |
| Syncope | 0 | 1 (16.7) | 0 | 1 (5.3) | 0 |
| Injury, poisoning, and procedural complications | 1 (16.7) | 0 | 2 (28.6) | 3 (15.8) | 0 |
| Vaccination complications | 0 | 0 | 2 (28.6) | 2 (10.5) | 0 |
| Bite | 1 (16.7) | 0 | 0 | 1 (5.3) | 0 |
| Contusion | 1 (16.7) | 0 | 0 | 1 (5.3) | 0 |
| Fall | 1 (16.7) | 0 | 0 | 1 (5.3) | 0 |
| Neoplasms benign, malignant, and unspecified (incl. cysts and polyps) | 2 (33.3) | 1 (16.7) | 0 | 3 (15.8) | 0 |
| Fibrous histiocytoma | 1 (16.7) | 0 | 0 | 1 (5.3) | 0 |
| Invasive ductal breast carcinoma | 1 (16.7) | 0 | 0 | 1 (5.3) | 0 |
| Mucinous breast carcinoma | 0 | 1 (16.7) | 0 | 1 (5.3) | 0 |
| Reproductive system and breast disorders | 1 (16.7) | 1 (16.7) | 1 (14.3) | 3 (15.8) | 0 |
| Metrorrhagia | 1 (16.7) | 0 | 1 (14.3) | 2 (10.5) | 0 |
| Cervical dysplasia | 0 | 1 (16.7) | 0 | 1 (5.3) | 0 |
| Endometriosis | 0 | 1 (16.7) | 0 | 1 (5.3) | 0 |
| Blood and lymphatic system disorders | 0 | 1 (16.7) | 0 | 1 (5.3) | 3 (42.9) |
| Anemia | 0 | 1 (16.7) | 0 | 1 (5.3) | 3 (42.9) |
| Cardiac disorders | 1 (16.7) | 0 | 0 | 1 (5.3) | 1 (14.3) |
| Palpitations | 1 (16.7) | 0 | 0 | 1 (5.3) | 0 |
| Tachycardia | 0 | 0 | 0 | 0 | 1 (14.3) |
| Immune system disorders | 1 (16.7) | 0 | 0 | 1 (5.3) | 0 |
| Hypersensitivity | 1 (16.7) | 0 | 0 | 1 (5.3) | 0 |
| Metabolism and nutrition disorders | 1 (16.7) | 0 | 0 | 1 (5.3) | 0 |
| Hyperphagia | 1 (16.70 | 0 | 0 | 1 (5.3) | 0 |
| Pregnancy, puerperium, and perinatal conditions | 0 | 1 (16.7) | 0 | 1 (5.3) | 0 |
| Subchorionic hematoma | 0 | 1 (16.7) | 0 | 1 (5.3) | 0 |
| Renal and urinary disorders | 1 (16.7) | 0 | 0 | 1 (5.3) | 0 |
| Urge incontinence | 1 (16.7) | 0 | 0 | 1 (5.3) | 0 |
| Vascular disorders | 1 (16.7) | 0 | 0 | 1 (5.3) | 0 |
| Internal hemorrhage | 1 (16.7) | 0 | 0 | 1 (5.3) | 0 |

^a^Number of participants who experienced at least one AE.
AE = adverse event; URTI = upper respiratory tract infection.

## Supplemental Table 5

Adverse events suggestive of upper respiratory tract infections occurring between baseline and the end of the follow-up period in healthy volunteers administered a single subcutaneous dose of AMY109 0.6–5.0 mg/kg (Part 1).

| **n (%)^a^** | **AMY109 dose, mg/kg** | | | | | **Placebo (n=18)** |
| --- | --- | --- | --- | --- | --- | --- |
|  | **0.6 (n=6)** | **2.0 (n=12)** | **3.5 (n=14)** | **5.0 (n=26)** | **Total (n=58)** |  |
| Any AE suggestive of URTI^b^ | 0 | 1 (8.3) | 4 (28.6) | 7 (26.9) | 12 (20.7) | 4 (22.2) |
| Any AE suggestive of treatment-related URTI^b^ | 0 | 0 | 0 | 0 | 0 | 0 |
| Infections and infestations | 0 | 1 (8.3) | 2 (14.3) | 6 (23.1) | 9 (15.5) | 3 (16.7) |
| Pharyngitis | 0 | 0 | 1 (7.1) | 4 (15.4) | 5 (8.6) | 1 (5.6) |
| URTI | 0 | 1 (8.3) | 1 (7.1) | 2 (7.7) | 4 (6.9) | 1 (5.6) |
| Tonsillitis | 0 | 0 | 1 (7.1) | 1 (3.8) | 2 (3.4) | 0 |
| Epiglottitis | 0 | 0 | 0 | 1 (3.8) | 1 (1.7) | 0 |
| Laryngitis | 0 | 0 | 0 | 1 (3.8) | 1 (1.7) | 0 |
| Rhinitis | 0 | 0 | 1 (7.1) | 0 | 1 (1.7) | 0 |
| Nasopharyngitis | 0 | 0 | 0 | 0 | 0 | 1 (5.6) |
| Sinusitis | 0 | 0 | 0 | 0 | 0 | 1 (5.6) |
| Respiratory, thoracic, and mediastinal disorders | 0 | 0 | 4 (28.6) | 1 (3.8) | 5 (8.6) | 2 (11.1) |
| Upper respiratory tract inflammation | 0 | 0 | 3 (21.4) | 0 | 3 (5.2) | 0 |
| Rhinorrhea | 0 | 0 | 1 (7.1) | 1 (3.8) | 2 (3.4) | 2 (11.1) |
| Productive cough | 0 | 0 | 1 (7.1) | 0 | 1 (1.7) | 0 |
| Vocal cord inflammation | 0 | 0 | 0 | 0 | 0 | 1 (5.6) |

^a^Number of participants who experienced at least one AE.
^b^Defined by appropriate medical review.
AE = adverse event; URTI = upper respiratory tract infection.

## Supplemental Table 6

Adverse events suggestive of infections occurring between baseline and the end of the follow-up period in healthy volunteers who were administered a single subcutaneous dose of AMY109 0.6–5.0 mg/kg (Part 1).

| **n (%)^a^** | **AMY109 dose, mg/kg** | | | | | **Placebo (n=18)** |
| --- | --- | --- | --- | --- | --- | --- |
|  | **0.6 (n=6)** | **2.0 (n=12)** | **3.5 (n=14)** | **5.0 (n=26)** | **Total (n=58)** |  |
| Any AE suggestive of infection^b^ | 1 (16.7) | 1 (8.3) | 4 (28.6) | 7 (26.9) | 13 (22.4) | 6 (33.3) |
| Any AE suggestive of treatment-related infection^b^ | 1 (16.7) | 0 | 0 | 1 (3.8) | 2 (3.4) | 2 (11.1) |
| Infection and infestations | 1 (16.7) | 1 (8.3) | 3 (21.4) | 6 (23.1) | 11 (19.0) | 5 (27.8) |
| Pharyngitis | 0 | 0 | 1 (7.1) | 4 (15.4) | 5 (8.6) | 1 (5.6) |
| URTI | 0 | 1 (8.3) | 1 (7.1) | 2 (7.7) | 4 (6.9) | 1 (5.6) |
| Tonsillitis | 0 | 0 | 1 (7.1) | 1 (3.8) | 2 (3.4) | 0 |
| Epiglottitis | 0 | 0 | 0 | 1 (3.8) | 1 (1.7) | 0 |
| Gastroenteritis | 0 | 0 | 1 (7.1) | 0 | 1 (1.7) | 0 |
| Herpes zoster | 0 | 0 | 0 | 1 (3.8) | 1 (1.7) | 0 |
| Laryngitis | 0 | 0 | 0 | 1 (3.8) | 1 (1.7) | 0 |
| Rhinitis | 0 | 0 | 1 (7.1) | 0 | 1 (1.7) | 0 |
| Tinea infection | 1 (16.7) | 0 | 0 | 0 | 1 (1.7) | 2 (11.1) |
| Nasopharyngitis | 0 | 0 | 0 | 0 | 0 | 1 (5.6) |
| Sinusitis | 0 | 0 | 0 | 0 | 0 | 1 (5.6) |
| Respiratory, thoracic, and mediastinal disorders | 0 | 0 | 4 (28.6) | 1 (3.8) | 5 (8.6) | 2 (11.1) |
| Upper respiratory tract inflammation | 0 | 0 | 3 (21.4) | 0 | 3 (5.2) | 0 |
| Rhinorrhea | 0 | 0 | 1 (7.1) | 1 (3.8) | 2 (3.4) | 2 (11.1) |
| Productive cough | 0 | 0 | 1 (7.1) | 0 | 1 (1.7) | 0 |
| Vocal cord inflammation | 0 | 0 | 0 | 0 | 0 | 1 (5.6) |
| Gastrointestinal disorders | 0 | 0 | 1 (7.1) | 0 | 1 (1.7) | 0 |
| Enterocolitis | 0 | 0 | 1 (7.1) | 0 | 1 (1.7) | 0 |

^a^Number of participants who experienced at least one AE.
^b^Defined by appropriate medical review.
AE = adverse event; URTI=upper respiratory tract infection.

## Supplemental Table 7

Adverse events suggestive of infections occurring between baseline and the end of the follow-up period in patients with endometriosis who were administered once-monthly subcutaneous doses of AMY109 0.8–5.0 mg/kg for 6 months (Part 2).

| **n (%)^a^** | **AMY109 dose, mg/kg** | | | | **Placebo (n=7)** |
| --- | --- | --- | --- | --- | --- |
|  | **0.8 (n=6)** | **2.0 (n=6)** | **5.0 (n=7)** | **Total (n=19)** |  |
| Any AE suggestive of infection^b^ | 2 (33.3) | 1 (16.7) | 1 (14.3) | 4 (21.1) | 1 (14.3) |
| Any AE suggestive of treatment-related infection^b^ | 0 | 0 | 0 | 0 | 0 |
| Infection and infestations | 2 (33.3) | 1 (16.7) | 1 (14.3) | 4 (21.1) | 1 (14.3) |
| Conjunctivitis | 1 (16.7) | 0 | 0 | 1 (5.3) | 0 |
| URTI | 0 | 0 | 0 | 0 | 1 (14.3) |
| Nasopharyngitis | 2 (33.3) | 1 (16.7) | 1 (14.3) | 4 (21.1) | 0 |
| Respiratory, thoracic, and mediastinal disorders | 1 (16.7) | 0 | 0 | 1 (5.3) | 0 |
| Rhinorrhea | 1 (16.7) | 0 | 0 | 1 (5.3) | 0 |

^a^Number of participants who experienced at least one AE.
^b^Defined by appropriate medical review.
AE = adverse event; URTI=upper respiratory tract infection.

## Supplemental Table 8

Adverse events suggestive of upper respiratory tract infections occurring between baseline and the end of the follow-up period in patients with endometriosis administered once-monthly subcutaneous doses of AMY109 0.8–5.0 mg/kg for 6 months (Part 2).

| **n (%)^a^** | **AMY109 dose, mg/kg** | | | | **Placebo (n=7)** |
| --- | --- | --- | --- | --- | --- |
|  | **0.8 (n=6)** | **2.0 (n=6)** | **5.0 (n=7)** | **Total (n=19)** |  |
| Any AE suggestive of URTI^b^ | 2 (33.3) | 1 (16.7) | 1 (14.3) | 4 (21.1) | 1 (14.3) |
| Any AE suggestive of treatment-related URTI^b^ | 0 | 0 | 0 | 0 | 0 |
| Infections and infestations | 2 (33.3) | 1 (16.7) | 1 (14.3) | 4 (21.1) | 1 (14.3) |
| URTI | 0 | 0 | 0 | 0 | 1 (14.3) |
| Nasopharyngitis | 2 (33.3) | 1 (16.7) | 1 (14.3) | 4 (21.1) | 0 |
| Respiratory, thoracic, and mediastinal disorders | 1 (16.7) | 0 | 0 | 1 (5.3) | 0 |
| Rhinorrhea | 1 (16.7) | 0 | 0 | 1 (5.3) | 0 |

^a^Number of participants who experienced at least one AE.
^b^Defined by appropriate medical review.
AE = adverse event; URTI = upper respiratory tract infection.

## Supplemental Table 9

Summary of AMY109 pharmacokinetic parameters.

| **Part** | **Cohort^a^** | **Sex** | **Dose** | **AUC_last_** | **T_max_** | **C_max_** | **t_1/2_** | **AUC_inf_** |
| --- | --- | --- | --- | --- | --- | --- | --- | --- |
|  |  |  | **mg/kg** | **Mean ± SD, day*μg/mL** | **Median (range), day** | **Mean ± SD, μg/mL** | **Mean ± SD, day** | **Mean ± SD, day*μg/mL** |
| 1-1 HV | A-1 | M | 0.6 | 211 ± 30.5^b^ | 7.0  (6.0–7.0)^b^ | 4.0 ± 0.8^b^ | 39.2 ± 6.5^b^ | 238 ± 35.3^e^ |
|  | A-2 | M | 2.0 | 684 ± 26.5^b^ | 7.0  (4.0–10.1)^b^ | 13.4 ± 0.6^b^ | 44.8 ± 5.1^b^ | 822 ± 39.7^e^ |
|  | A-3 | M | 5.0 | 1600 ± 206^b^ | 10.0  (4.0–14.1)^b^ | 32.2 ± 5.1^b^ | 41.3 ± 8.0^b^ | 1890 ± 246^b^ |
|  | B-1 | M | 2.0 | 490 ± 225^b^ | 8.5  (4.0–10.0)^b^ | 11.2 ± 3.7^b^ | 42.3 ± 10.2^e^ | 642 ± 177^f^ |
|  | B-2 | M | 5.0 | 1400 ± 286^b^ | 8.5 (4.0–21.0)^b^ | 27.0 ± 5.6^b^ | 55.8 ± 34.1^b^ | 1610 ± 369^e^ |
|  | C-1 | F | 5.0 | 1330 ± 330^b^ | 10.0  (10.0–21.1)^b^ | 23.5 ± 6.8^b^ | 39.0 ± 5.0^b^ | 1480 ± 379^e^ |
| 1-2 HV | D-1 | F | 3.5 | 1120 ± 225^b^ | 10.5  (5.0–21.1)^b^ | 18.0 ± 4.9^b^ | 48.4 ± 9.5^b^ | 1300 ± 298^b^ |
|  | E-1 | M | 3.5 | 1170 ± 216^c^ | 7.0  (3.0–14.1)^c^ | 19.9 ± 3.2^c^ | 46.1 ± 8.7^c^ | 1360 ± 281^c^ |
|  | E-2 | M | 5.0 | 1670 ± 309^c^ | 10.0  (3.0–14.1)^c^ | 27.2 ± 7.5^c^ | 41.9 ± 5.8^c^ | 1890 ± 308^c^ |
| 2  Pt | 1 | F | 0.8 | 110 ± 24.5^b^ | 13.4  (3.0–25.9)^b^ | 4.9 ± 1.0^b^ | – | – |
|  | 2 | F | 2.0 | 225 ± 57.1^b^ | 10.5 (5.9–15.1)^b^ | 10.0 ± 3.0^b^ | – | – |
|  | 3 | F | 5.0 | 644 ± 78.9^d^ | 8.0  (6.0–15.0)^d^ | 26.5 ± 3.5^d^ | – | – |

^a^Cohorts A-1, A-2, and A-3: 0.6, 2.0, and 5.0 mg/kg in Asian male HVs, respectively; cohorts B-1 and B-2: 2.0 and 5.0 mg/kg in white male HVs, respectively; cohort C-1: 5.0 mg/kg in Asian female HVs; cohort D-1: 3.5 mg/kg in white female HVs; cohorts E-1 and E-2: 3.5 and 5.0 mg/kg in Asian male HVs; cohorts 1, 2 and 3: 0.8, 2.0, and 5.0 mg/kg in patients with endometriosis, respectively.
^b^n=6.
^c^n=8.
^d^n=7.
^e^n=5.
^f^n=4.
HV=healthy volunteer, Pt= patient, M=male, F=female, AUC_inf_ = area under the plasma concentration versus time curve extrapolated to infinity; AUC_last_ = AUC to the last measurable concentration; C_max_ = peak plasma concentration; SD = standard deviation; T_max_ = time to C_max_; t_1/2_ = elimination half-life.

## Supplemental Table 10

Effects of race on AMY109 pharmacokinetics in healthy volunteers (Part 1).

| **Parameter** | **Race** | **n** | **GLSM^a^** | **GLSM Asian:White ratio (90% CI)^a^** |
| --- | --- | --- | --- | --- |
| **AMY109 2.0 mg/kg** | | | | |
| AUC_0–t_, day*μg/mL | White | 6 | 417 | – |
|  | Asian | 6 | 684 | 1.64 (0.95–2.82) |
| AUC_0–t_/dose, mg/kg | White | 6 | 207 | – |
|  | Asian | 6 | 342 | 1.65 (0.96–2.84) |
| AUC_inf_, day*μg/mL | White | 4 | 623 | – |
|  | Asian | 5 | 821 | 1.32 (1.03–1.68) |
| AUC_inf_/dose, mg/kg | White | 4 | 309 | – |
|  | Asian | 5 | 411 | 1.33 (1.04–1.70) |
| C_max_, μg/mL | White | 6 | 10.7 | – |
|  | Asian | 6 | 13.4 | 1.24 (0.97–1.60) |
| C_max_/dose, mg/kg | White | 6 | 5.3 | – |
|  | Asian | 6 | 6.7 | 1.25 (0.98–1.61) |
| **AMY109 5.0 mg/kg** | | | | |
| AUC_0–t_, day*μg/mL | White | 6 | 1370 | – |
|  | Asian | 6 | 1590 | 1.16 (0.96–1.40) |
| AUC_0–t_/dose, mg/kg | White | 6 | 274 | – |
|  | Asian | 6 | 319 | 1.17 (0.97–1.41) |
| AUC_inf_, day*μg/mL | White | 5 | 1570 | – |
|  | Asian | 6 | 1870 | 1.19 (0.97–1.47) |
| AUC_inf_/dose, mg/kg | White | 5 | 313 | – |
|  | Asian | 6 | 376 | 1.20 (0.97–1.48) |
| C_max_, μg/mL | White | 6 | 26.5 | – |
|  | Asian | 6 | 31.9 | 1.20 (0.98–1.47) |
| C_max_/dose, mg/kg | White | 6 | 5.3 | – |
|  | Asian | 6 | 6.4 | 1.21 (0.99–1.48) |

^a^The GLSMs, ratios of GLSMs, and corresponding CIs were obtained by taking the exponential of the LSM, differences in LSMs, and corresponding CIs on the natural log (ln) scale.
AUC_0–t_ = area under the plasma concentration-time curve from time 0 to the time of the last quantifiable concentration; AUC_inf_ = area under the plasma concentration versus time curve extrapolated to infinity; CI = confidence interval; C_max_ = peak plasma concentration; GLSM = geometric least squares mean; LSM = least squares mean.

## Supplemental Table 11

Mean monthly visual analog scale scores of dysmenorrhea and non-menstrual pelvic pain at baseline and different timepoints during treatment and at the end of follow up in patients with endometriosis administered once-monthly subcutaneous doses of AMY109 0.8–5.0 mg/kg for 6 months (Part 2).

| **VAS (mm)** | **AMY109 dose, mg/kg** | | | **Placebo (n=7)** |
| --- | --- | --- | --- | --- |
|  | **0.8 (n=6)** | **2.0 (n=6)** | **5.0 (n=7)** |  |
| Mean score of dysmenorrhea^a^ | | | | |
| Baseline | 63.4 | 38.0^c^ | 40.8 | 36.8 |
| Dose 3^b^ | 45.7 (−11.0)^c^ | 30.5 (−10.7)^c^ | 26.9 (−14.3)^e^ | 21.5 (−14.4)^e^ |
| Dose 6 (treatment end)^b^ | 42.9 (−6.6)^d^ | 28.4 (−18.0)^d^ | 23.0 (−16.9)^e^ | 18.0 (−17.9)^e^ |
| Follow-up^b^ | 46.5 (−3.0)^d^ | 27.8 (−19.9)^d^ | 18.4 (−21.5)^e^ | 17.8 (−21.0)^e^ |
| Mean score of non-menstrual pelvic pain^f^ | | | | |
| Baseline | 32.3 | 27.4 | 18.5 | 13.3 |
| Dose 3^b^ | 22.5 (+2.3)^c^ | 26.8 (−1.3) | 11.8 (−6.7) | 8.7 (−4.6) |
| Dose 6 (treatment end)^b^ | 21.5 (−0.4)^d^ | 20.7 (−2.0)^g^ | 10.3 (−10.0)^e^ | 10.5 (−2.8) |
| Follow-up^b^ | 20.6 (−1.3)^d^ | 7.1 (−4.5)^g^ | 8.1 (−12.2)^e^ | 6.1 (−7.2) |

^a^The days with no uterine bleeding were excluded from analysis.
^b^Difference in mean VAS from baseline is presented in parentheses.
^c^n=5.
^d^n=4.
^e^n=6.
^f^The days with uterine bleeding were excluded from analysis.
^g^n=3.
VAS = visual analog scale.

## Supplemental Table 12

Modified Biberoglu and Behrman Scale scores of dysmenorrhea and non-menstrual pelvic pain occurring at baseline and different time points during treatment and at the end of follow-up in patients with endometriosis who were administered once-monthly subcutaneous doses of AMY109 0.8–5.0 mg/kg for 6 months (Part 2).

| **mBBS** | **AMY109 dose, mg/kg** | | | **Placebo (n=7)** |
| --- | --- | --- | --- | --- |
|  | **0.8 (n=6)** | **2.0 (n=6)** | **5.0 (n=7)** |  |
| Mean score of dysmenorrhea^a^ | | | | |
| Baseline | 2.0 | 1.3^c^ | 1.3 | 1.3 |
| Dose 3^b^ | 1.5 (–0.3)^c^ | 1.1 (–0.4)^c^ | 0.9 (–0.4)^e^ | 0.9 (–0.4)^e^ |
| Dose 6 (treatment end)^b^ | 1.2 (–0.4)^d^ | 1.2 (–0.5)^d^ | 0.9 (–0.5)^e^ | 0.6 (–0.6)^e^ |
| Follow-up^b^ | 1.2 (–0.4)^d^ | 1.0 (–0.7)^d^ | 0.7 (–0.6)^e^ | 0.6 (–0.8)^e^ |
| Mean score of non-menstrual pelvic pain^f^ | | | | |
| Baseline | 1.0 | 0.9 | 0.7 | 0.6 |
| Dose 3^b^ | 0.6 (–0.1)^c^ | 0.8 (–0.1)^c^ | 0.4 (–0.4) | 0.4 (–0.2) |
| Dose 6 (treatment end)^b^ | 0.6 (–0.1)^d^ | 0.9 (0.2)^g^ | 0.4 (–0.4)^e^ | 0.4 (–0.2) |
| Follow-up^b^ | 0.6 (–0.1)^d^ | 0.2 (–0.1)^g^ | 0.3 (–0.5)^e^ | 0.2 (–0.4) |

^a^The days with no uterine bleeding were excluded from analysis.
^b^Mean change in mBBS score from baseline is presented in parentheses.
^c^n=5.
^d^n=4.
^e^n=6.
^f^The days with uterine bleeding were excluded from analysis.
^g^n=3.
mBBS = modified Biberoglu and Behrman Scale.

# Supplemental Figures

## Supplemental Fig. 1.

Participant flow diagram. All the enrolled participants were included in the safety analysis, the primary endpoint of the study. AE, adverse event.


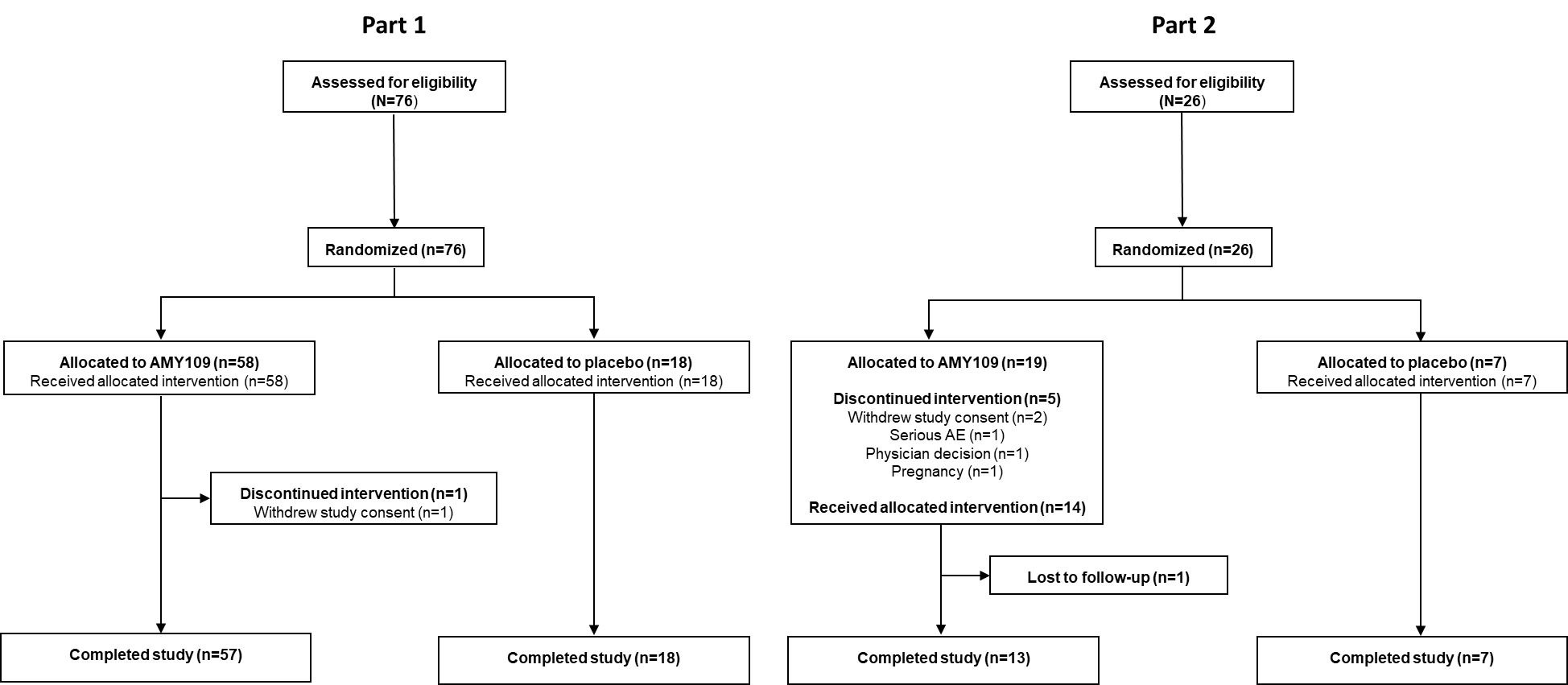


## Supplemental Fig. 2.

Correlation analysis between adverse events (AEs) and pharmacokinetics for (A) AUC vs AEs in Part 1, (B) C_max_ vs AEs in part 1, (C) AUC vs AEs in Part 2, (D) C_max_ vs AEs in Part 2, (E) AUE vs AEs in Part 1, (F) E_max_ vs AEs in Part 1, (G) AUE vs AEs in Part 2, and (H) AUE vs E_max_ in Part 2. AUC, C_max_, AUE, and E_max_ values were estimated up to the onset of AEs. The grade of AEs was set at Grade >1. Placebo data were excluded for AUE and E_max_ because the free IL-8 levels were calculated using AMY109 concentrations and total IL-8 levels. Logistic regression analyses are shown by the regression curve and 95% confidence interval in each panel. AUC = area under time-plasma AMY109 concentration curve; C_max_ = maximum plasma AMY109 concentration; AUE = area under time-free IL-8 (effect) concentration curve; E_max_ = maximum free IL-8 (effect) level; IL = interleukin.


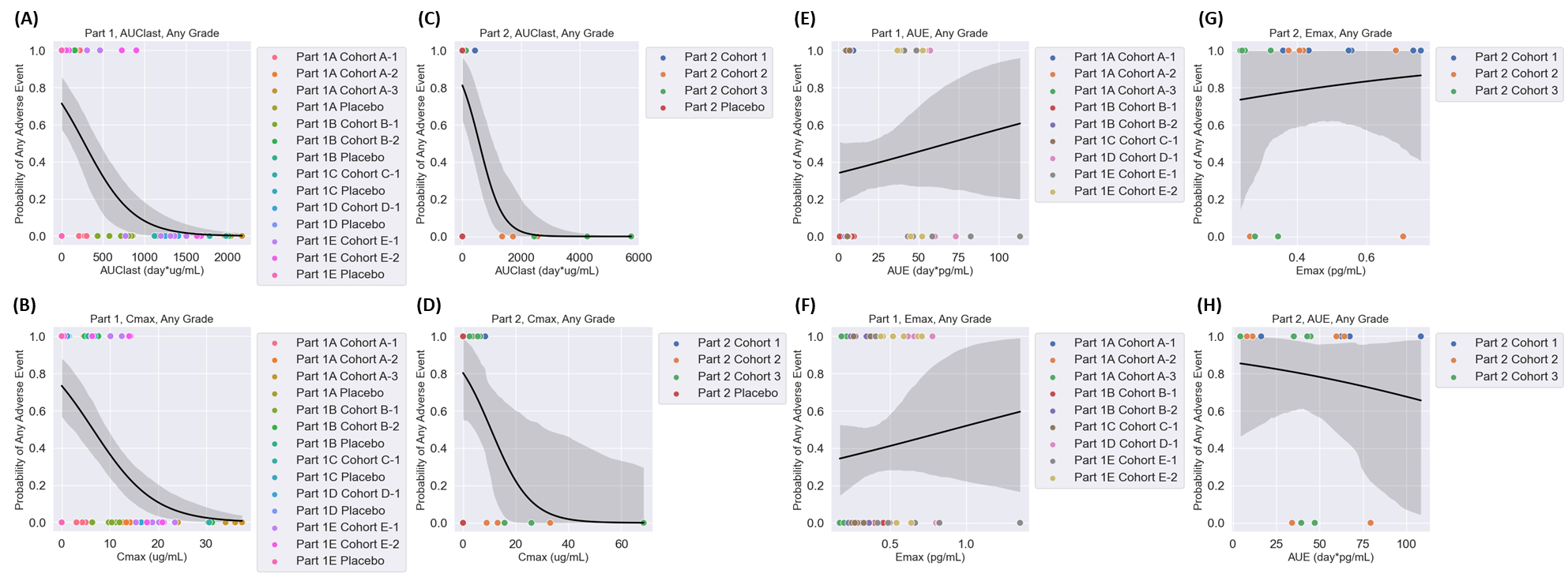


## Supplemental Fig. 3.

Box and whisker plots showing analgesic medication use in patients with endometriosis who received once-monthly subcutaneous doses of (A) AMY109 0.8 mg/kg, (B) AMY109 2.0 mg/kg, (C) AMY109 5.0 mg/kg, or (D) placebo for 6 months (Part 2). The whiskers denote the maximum and minimum values, the ends of the box the first and third quartiles, the horizontal line in the box the median value, the diamond the mean value, and the colored dots the individual values. FU = follow-up.


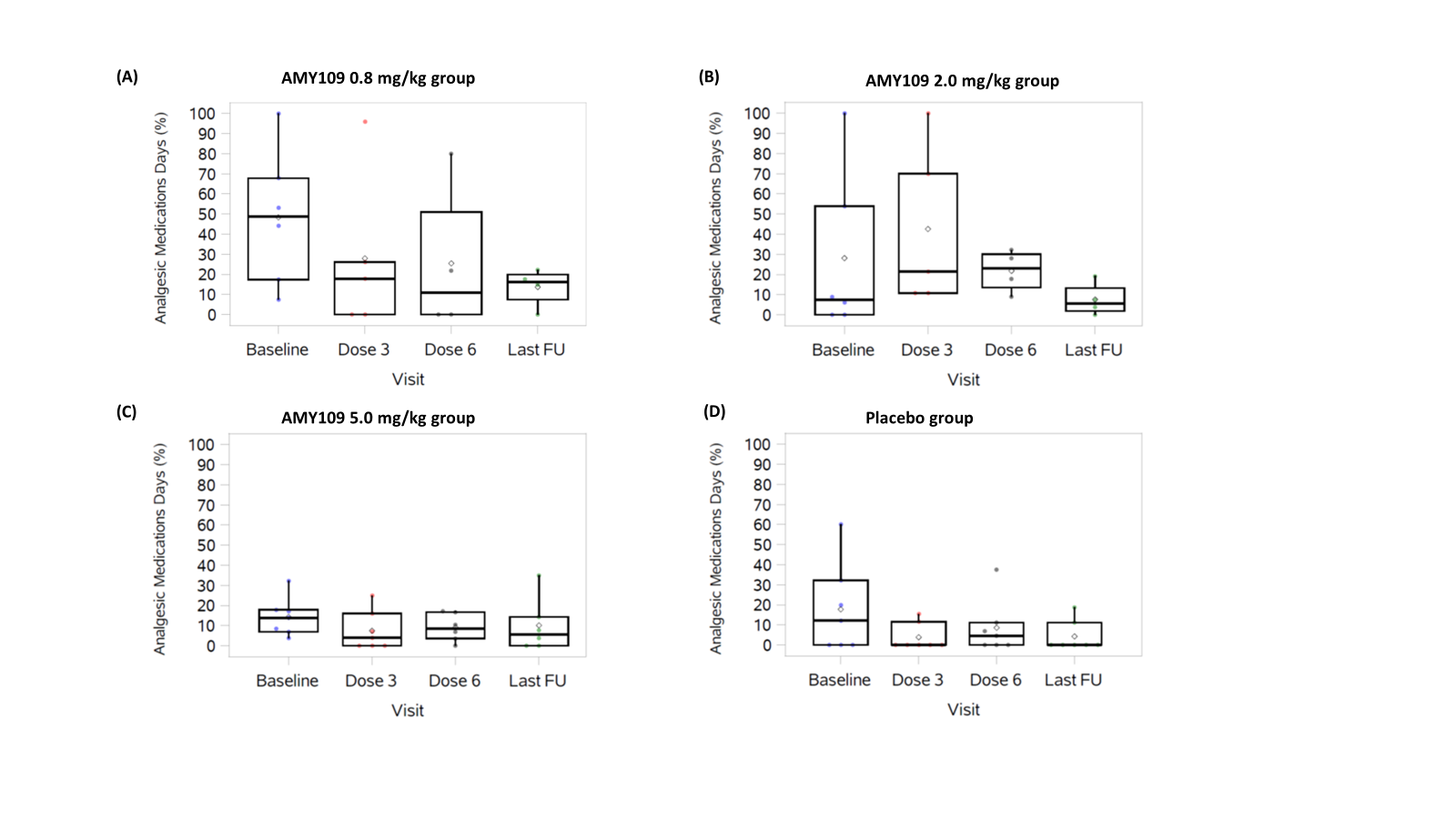

Supplement: Supplementary Material [file mmc1.docx]
